# On the Observation of Nature in the Study and Treatment of Disease

**Published:** 1847-01

**Authors:** Andrew Combe


### PART THIRD.

### CONTRIBUTIONS TOWARDS THE ADVANCEMENT

### OF THE

### Natural History and Treatment of Diseases.

---

### I. ON THE OBSERVATION OF NATURE IN THE STUDY AND TREATMENT OF DISEASE.

BY ANDREW COMBE, M.D.

(*In a Letter to John Forbes, M.D., F.R.S.*)

Edinburgh, December 11, 1846.

My Dear Sir.—Dr. Symonds's able letter in the last number of your Review reminds me forcibly of an occurrence which took place during my graduation here, upwards of twenty years ago. I had written a thesis, the chief purpose of which was to show that the dyspeptic symptoms in hypochondriasis are frequently *secondary*, both in importance and in the order of their appearance, and that while they may remain intractable under a treatment directed exclusively to the digestive organs, they often yield with facility after the removal of the *primary* cerebral and mental affection which originated them. Such was my doctrine. Judge then of my surprise when gravely informed by the late venerable professor to whose perusal my thesis was intrusted, that it was a very good dissertation, but that I had certainly committed an important practical error in maintaining that *dyspepsia was never present in hypochondriasis!* On attempting to explain that I had broached no such opinion, the worthy professor cut me short by adding, with a bland and forgiving smile, that farther explanation was needless, as he felt sure I would profit by the hint he had thrown out. At that time such occurrences were new to me, and being taken by surprise I could only make my bow and retire to digest the remark at leisure and make the best of it.

Further experience, however, has since convinced me that in the scientific and literary worlds, it is by no means so rare a thing as I had imagined, to be criticised for the advocacy of opinions which were never entertained, and by critics whose views on all essential points are nearly the same as our own. Indeed, I believe that this very assumption of the existence of a contradiction where in reality there is little or none, constitutes one of the most serious impediments to the discovery and diffusion of new truths. Profiting by the lesson, it seems to me a wise precaution, before proceeding to refute a supposed antagonist by either argument or ridicule, to ascertain very clearly whether a difference exists, and if so, in what it really consists. If Dr. Symonds had acted on this principle, before writing his letter against excessive trust in Nature, I think he would have discovered that he was about to take much trouble to refute errors which were mostly imaginary; and that when he condemned as wholly inert and absurd, plans of treatment conducted in accordance with Nature, he had formed an idea of them in his own mind, not only unwarranted by the published letters, but directly opposed to their spirit, and as such, repudiated not less by our principles than by his own. In fact, wherever Dr. Symonds observes and describes without any reference to theory, and wherever his practical views are fairly tried by the standard we recommend, there is found to be far less divergence between them than he seems to believe, or than any one would suppose from the mere perusal of his letter. It is true that he very rarely makes any special reference to the standard of Nature, but he has too much acuteness of observation and shrewd sagacity not to make frequent unconscious approaches to it in practice, and whenever he does so, there is generally harmony and almost never contradiction between us. If he were more familiar with the study of Nature as an aim, he would see that most of the opinions which he denounces are the unsubstantial offspring of his own misapprehension, and as such need not cause him any uneasiness, since they cannot continue their existence in his mind beyond the lifetime of their parent-error. But to come to proofs.

After alluding in general terms to the recent articles in your Review,—mine among the number—on the better observation of Nature in the study and treatment of disease, Dr. Symonds proceeds to say, " The question before us is this: *whether it is safer to leave diseases to their own course, or to interpose what we call remedies?* And really this," he continues, "*is a very frightful question* to be asking at the present state of the world's history, when medicine has been proposed as an art two thousand years and more." (p. 558.)

Concurring as I do most cordially in this latter sentiment, it seems to me strange that this very frightfulness should not have suggested a doubt to Dr. Symonds, whether he had not greatly *misapprehended*, and consequently unintentionally *misrepresented* the question that was really before him. If such a doubt had suggested itself, and he had thereby been led to inquire more carefully, all his fears would have vanished, for he would have seen that such was NOT the question which was before him, and that were it possible for a question so inherently absurd to be ever seriously put, there would be *all but unanimity* in the answer which it would meet with from the profession and from the public. It is true that in his letter, Dr. Symonds *seems* to express a contrary conviction; but so far from that conviction being well founded, I feel assured that it would puzzle him to produce one medical man who would, in his sound senses, affirm that it is "safer to leave diseases to their own course than to interpose remedies," and he has been led to an opposite conclusion only by adopting the mistaken impression, that treatment conducted in accordance with the laws and intentions of Nature (which, for the sake of brevity, I shall call the *natural treatment*) is *identical with no treatment at all*—a construction at once palpably erroneous and unwarranted. So common, however, is this misconstruction among those who have not thought on the subject, that it is everywhere the first objection made to natural treatment; and accordingly in your April Number I protested against it by anticipation (at p. 508), and even accused *you* of "an omission in your article which will tend to the diffusion of a misapprehension already too prevalent in a point of vital importance, and of having unguardedly used expressions which, *per se*, might be held to justify it." Again, at page 513, I recurred to the subject, and said, "By thus insisting on the necessity of a more complete and faithful observation of the course of Nature, and of acting more systematically under her guidance, *I am far from meaning that we are to sit with our hands across and allow things to take their own way.* So far from it, it is certain that the principle I inculcate would *demand more watchfulness, and give room for a nicer exercise of judgment, and a more consistent, and, I believe, successful treatment;*" and yet Dr. Symonds argues as if my object was to " leave things to take their own way"—a proceeding which he cannot condemn more strongly than I do.

These references may perhaps suffice to show that the question, as stated by Dr. Symonds, is neither the question really before the profession, nor that warranted by the spirit and substance of the article on which he comments; and it will not be difficult to show farther that, as already remarked, his practical views differ little in substance from those deducible from the principles to which he imagines himself to be so strongly opposed. To put this in a clear light, let us take two or three of the examples selected by himself as supposed contrasts to the practice he believes us to advocate: "If one were at a loss," he says, "for instances in which *art* interfered with advantage when *Nature* was doing nothing, or rather doing mischief, I doubt if any instance could be more striking than the cure and relief of many of the diseases of children by lancing the gums. On the expectant method a child may, after many days of suffering, and after incurring perils in various organs, at last recover when the tooth has pushed its way into the light, but all the pain and jeopardy might have been spared by one or two free incisions." (p. 561.)

Now the whole hostile force of this illustration depends on the question of fact, whether the contrast which Dr. Symonds supposes to exist between a treatment directed with a view to "*aid the efforts of Nature*" and what he calls "*art*" is real or imaginary? If he is right in assuming (*which he does throughout his whole letter*) that a treatment conducted in accordance with the laws of Nature consists in *doing absolutely nothing*, or, as he phrases it, in "leaving diseases to follow their own course," then the above example is at once pregnant, instructive, and conclusive against us. But if, on the other hand, it is, *as we contend*, an utter confusion of language and ideas to speak of two things so essentially different as if they were one and the same, the example ceases to have either meaning or value, except as showing how much a man of strong sense may be blinded by a preconceived notion. In this very case of teething it so happens that the correct application of the principles of natural treatment would lead to the use of the very same means which Dr. Symonds states from experience to be alone appropriate. In teething, *Nature's efforts* are directed to effecting a passage outwards for the advancing tooth. If, from any circumstance, she proves unable for the effort, sound principle would assuredly direct us to *aid her* in it, and remove the obstacles which obstruct her progress; and I am at a loss to know what more rational or direct *aid* we could lend her in her own way, than making the opening through the gum, which she has failed to accomplish for herself! In the outset of the letter which Dr. Symonds criticises, I state that it is the business of the physician to "take special care neither to counteract her (Nature's) efforts, nor to substitute another *method of cure* for hers, *unless when we have positive evidence that the vis medicatrix,* JUDICIOUSLY AIDED BY US, *will prove unavailing.*" (p. 506.) To apply this principle to the case before us, it seems to me to indicate that when Nature is perfectly able to make a passage for the tooth, we ought not to counteract or disturb her efforts, but that *as soon as we have evidence that her attempts will prove unavailing, and that danger will arise, we ought to come to her assistance* and divide the gum for her. In what respects Dr. Symonds's practical views differ from the above, he himself would be puzzled to tell.

Perhaps, however, the best way of exhibiting the real difference between a natural system of treatment and "leaving diseases to follow their own course," which Dr. Symonds seems to consider as identical with it, will be to take the example of a deep sabre cut, the progress and results of which are visible, and consequently admit of no dispute. If the wound be left to "*follow its own course*," and any large vessels happen to be divided, the efforts of Nature to arrest the hemorrhage by the retraction of the artery and the formation of a coagulum will prove unavailing, and the patient will bleed to death. If, however, only small vessels be implicated, the efforts of Nature will prove successful, and after a time the bleeding will cease. But from the wound being left to follow its own course, it will remain with its lips apart, and if it heal at all it will be only by the slow and insecure process of granulation. Such is the one method. If, on the other hand, we act on our principle of *assisting Nature,* the moment we find her efforts to arrest the hemorrhage unavailing, we shall come to her aid either by compressing or tying the artery. Having previously studied the order of Nature in the healing of wounds, we know that if their cut sides be brought into even contact, and the part kept quiet, union by the first intention will follow in a few days, *because the conditions required by Nature will then have been fulfilled.* Such accordingly is the plan of treatment really indicated by our principles, and its success justifies the unanimity with which it is acted upon. Why then should Dr. Symonds frighten himself about a phantom when his realities are the same as our own?

In like manner, take a fracture as an illustration. From observing Nature, we know that when a bone is broken increased vascular action must take place in the injured parts before reunion can be effected; that if it be *defective* in amount, the broken ends may remain in contact for months without uniting; and that if *excessive,* suppuration may ensue and also retard the union. Guided by this knowledge, the surgeon endeavours to second Nature's intentions by securing the limb in a proper position, keeping it motionless, and using any means which may be required to keep the reaction *at its proper pitch.* Having done so, he leaves Nature to complete her own work, and in due time has his reward. When the local action is too feeble, he, with the like intent of *aiding her,* rubs the ends of the bone rudely together or introduces a seton, and thus often succeeds in rousing Nature to successful exertion. If again the inflammation runs so high as to threaten suppuration, the surgeon, knowing that its occurrence would defeat her object, uses means to allay it, and often succeeds. Why then does Dr. Symonds speak as if all this were the same as leaving the fracture to "follow its own course," when it is precisely the treatment which he himself would pursue?

It may seem supererogatory to adduce seriously so much evidence in support of a proposition, which when thus placed in its true light is almost self-evident. But the improvement of medicine is so much retarded by the prevailing but most unfounded assumption, that practice directed by the indications of and in accordance with the laws of Nature, necessarily excludes all active means, and consists literally in *doing nothing,* and practical men often attach so little importance to the advantages derivable from an intelligent co-operation with Nature, that it becomes indispensable to direct attention to the subject by every means in our power. So impressed is Dr. Symonds, in common with Dr. Mayo and many other men, with this most erroneous conviction, that it pervades almost every paragraph of his letter. Hence we find him saying, at p. 559—"But to come to the cases in which it is especially needful to have our minds made up as to the right course of practice.—I mean the acute visceral inflammations—when I become satisfied that these diseases *may be left to pursue their own way,*" (always the same phantom,) "my belief will have suffered a more complete *bouleversement* that it has ever yet undergone." To me it seems strange that Dr. Symonds should have ever penned such a sentence; and had he not been almost haunted by the error of supposing a natural treatment to be identical with doing nothing, the inherent absurdity of the proposition that acute inflammations were to be left to pursue their own way would certainly have led him to suspect that he had fallen into some unaccountable mistake in believing it to be advocated in the articles on which he was commenting. A natural system of treatment does not by any means exclude activity of practice, *where it is clearly called for to promote recovery or relieve suffering.* All that it provides against is the *abuse* of active interference, where *assisted Nature* would have effected the object with greater safety to the constitution. It is, perhaps, in acute visceral inflammations, more than in other diseases, that heroic treatment has been most indiscriminately used, and although a great improvement in their treatment has taken place within the last twenty years, it still remains for consideration, whether the activity of our interference may not be reduced still farther with advantage to the patient. Even in pneumonia, in which, till lately, every body believed very active treatment indispensable, we now find not only that a fair proportion of cures takes place under homœopathic treatment, but that, according to Dr. Balfour's statement in your last Number, the allopathic Skoda even "*considers the great advantage of not bleeding to be* THE SPEEDY *recovery.*" (p. 591.) In like manner, Dr. Marshall Hall, whom nobody will accuse of inert practice, when speaking of the treatment of pleurisy in the Parisian hospitals, which he at first ridiculed as "trifling," honestly confesses that "*where the treatment of pleuritis consists greatly in the application of mere cataplasms, a post-mortem examination in this disease is scarcely to be obtained, so generally do the patients recover.*" (Hall's Observations in Medicine, p. 68.) I do not say that these statements are sufficient to condemn active interposition *in all cases* of pneumonia and pleurisy, but they certainly tend to show that the necessity for its being carried so far as is usually done, is by no means so clear and urgent as represented by Dr. Symonds, and that bloodletting, for example, might sometimes be beneficially restricted in frequency and extent, and used with more discrimination and a sounder appreciation of Nature's requirements, than it is at present by the mass of practitioners.

From some of Dr. Symonds's general remarks as well as from his cases, there is reason to infer that, practically at least, his opinions are not so different as he imagines from those which we really advocate. After stating how strange it would be if there was no such thing as an Art of Medicine, (which on the *do-nothing* principle there could not be) he explains with equal acuteness and truth, that "*Art* after all is but *Nature in a new form—a fresh arrangement of the forces of Nature compelling them to work under new conditions.*" Now this is precisely the doctrine which we have been all along advocating; and all that we insist upon is, that we should carefully observe Nature for the purpose of discovering which mode of rearranging her forces is the best calculated to insure a speedy and safe recovery.—Here, then, we once more practically agree with Dr. Symonds.

Forgetting his own definition of Art and still haunted by the deceiving phantom, Dr. Symonds next refers to the cures effected by hygienic treatment without the aid of drugs. He admits that such things do happen, but says, "Allowing this to be true, we must nevertheless perceive that they *are interpositions of art.* If a patient is confined to bed, when, but for orders, he would be sitting up or walking about; if he is restricted to bread and water or milk, when otherwise he would be living as usual; if a particular temperature of his room is maintained &c. &c. and he gets well—his case surely ought not to be adduced as an example of the *curative powers of Nature.* There has been a decided *interference of Art.*" (p. 562.) Most certainly there has been both a decided and a wise interference of art, but of an art which was clearly our old friend "Nature in a new form," with her old forces "working under new conditions;" and the strange thing is, that Dr. Symonds should not recognize her features under so slight a disguise. His whole difficulty would vanish if he would keep in mind his own definition of art, and open his eyes to the fact that *even by his own statement,* the said hygienic treatment does certainly *not* consist in "leaving diseases to follow their own way." It is quite true that such cures as he supposes are not examples of the curative powers of *unaided* Nature, but the great aim of the treatment we advocate is to *aid her efforts as long as they promise to be successful when so aided, and only to substitute other methods when hers prove unavailing.* If true art is really Nature in another form, it becomes as clear as the sun at noon-day that recovery in the circumstances he supposes is as directly an example of the *curative powers of Nature* as the union of the fractured bone after art has made a fresh arrangement of her forces, by placing their ends in contact and preventing all disturbing motion or inflammation. Surely it is *not* art that actually solders together the broken fragments by extraneous cement, and just as little is it art which removes an internal inflammation which it can neither see nor touch. In both instances, Nature is in truth the curative agent; and all that art can do, or ought to attempt, is as far as possible to fulfil the conditions which observation shows to be most favorable for the success of her efforts.

Dr. Symonds refers to the operation for strangulated hernia and the tying of arteries as conclusive "instances of *Art versus Nature*." But both are palpably examples of art acting successfully on the previous indications of Nature. If an artery is divided, and its natural power of retraction proves insufficient to arrest the hemorrhage, and permit a coagulum to be formed as a preliminary to the obliteration of its canal, what does the ligature do if not the very thing which unaided Nature tried in vain to accomplish? In like manner in hernia, Nature continues to propel the contents of the bowel and carry on its circulation and vital action till, in consequence of a physical obstacle which she cannot overcome, her efforts prove fruitless. But, as stated in my former letter, when an impediment or obstruction comes in her way, it is the direct business of the practitioner to remove it; and hence, so far from objecting to an operation *when milder means cannot be found*, he would, on the principles of the natural treatment, at once agree with Dr. Symonds in resorting to it. No doubt the surgeon's knife is the instrument by which the impediment to the return of the bowel is removed, but surely it has been used to fulfil the indications of Nature, and it is Nature under the new arrangement of her forces which completes the work. If the objectors to the natural system of treatment will only dismiss from their minds the false notion that it means "*doing nothing*," they will at once perceive that there is no incompatibility whatever between it and the employment of even the most active interpositions of art, when necessary to secure in any individual case that arrangement of Nature's forces which is most likely to lead to recovery. All that is required, is that such active means should be resorted to only *when, where*, and *in the manner, indicated by the close observation of Nature's operations*, and not, as so often happens, from mere habit, tradition, caprice, or—worst of all—in the form of medicines given as the means of securing an adequate remuneration for attendance.

Another reason which shows that Dr. Symonds is practically a greater believer than he imagines in the principles of the *natural treatment* of disease, will be found towards the end of his letter, where he says, "I am not fond of arguments from final causes; but can it be doubted that the various medicines we possess were, as such, *a part of the plan of the universe designed to have a relation to morbid states of living organisms* as much as esculent matters to healthy conditions?" To this query every rational mind will concur in answering, with Dr. Symonds himself, that no doubt whatever can be entertained on the subject. But just as little can it be doubted that, in accordance with the plan of Nature, such medicines should be used only in "those morbid states" to *which they have the designed relation*, instead of being given, as they *often* are, with no definite aim, and for no intelligible reason, except compliance with custom. If we take quinine as a fair specimen of this class of medicines, and as designed by Nature to "have a certain relation to the morbid state" of ague, is it not self-evident that its exhibition would be included under the head of a natural system of treatment of that disease, and that having due regard to the observation of Nature, we should further be prompted to discover and remove the local causes by which the ague had been produced, and by which recovery was still impeded? It is known, for instance, that removal to a healthy locality will often suffice to put an end to the fits; and it may fairly be asked, would not removing the *cause from the patient* act in the same way as removing *the patient from it?* On the more common plan, however, of trusting too exclusively to medicines alone for the cure of disease, the continued operation of the cause, and the advantages which may be derived from a new and more salubrious arrangement of the forces of Nature in facilitating recovery and preventing the occurrence of disease, are apt to be too much overlooked.

"But," says Dr. Symonds, "what shall we say of fevers and the exanthemata? Only what nine-tenths of eclectics would affirm, viz., that the less heroically they are treated the better." Here then is still another proof that Dr. Symonds is not so wide apart from us as he imagined. *Fever and exanthemata are precisely the class of diseases the natural history of which is best known,* and on the right indications in which a sound physiology throws most light; and there is every reason to believe that in proportion as the natural history of other diseases becomes better understood, their treatment also will become less heroic and more accordant with the laws of Nature than at present, and, as a consequence, *more successful.*

I scarcely need say that in going into this subject at so much greater length than I intended, I am actuated by no feeling of disrespect towards Dr. Symonds, whose zeal and services and kindly intentions towards myself I fully appreciate. But I have given a prominence to his remarks because his character and position give them weight, and because, in this instance, he represents a large and influential class in the profession, whose objections to our views, although apparently formidable, are, from the very same cause, equally unreal as his own. In the 'Medical Gazette' Dr. Mayo, among others, took much trouble to refute errors, most of which consisted of misapprehensions of his own; and since the appearance of the articles in your Review, I have heard of various conversational *demolitions* of the supposed system of doing-nothing, by friends who, on farther inquiry as to the reality of the alleged opinions, found to their own surprise that they had expended their blows on the windmill when they believed they had been fighting a giant. Having adopted their own practical views merely as the results of experience or of previous teaching, they had failed to discover sooner that in their general bearing they were to a considerable extent coincident with those of a natural system of treatment; and hence, from pure misapprehension, they had attributed to me fancies which, in common with themselves, I wholly repudiated as too frightful to be believed.

If the subject under discussion had been one of a purely personal nature, I should not have occupied a single page of your Review with the correction of any of the mistakes which have been committed on either side. But where the application of an important scientific principle is concerned, and its soundness or unsoundness is calculated powerfully to affect the progress of an art bearing directly on human life and human happiness, personal considerations sink into insignificance, and it becomes a duty to point out every source of error, or of even apparent contradiction, which may come under our notice. It is with this purpose alone I have ventured to trouble you with these remarks, and I feel assured that Dr. Symonds will regard them as applied, not to himself, but to the subject. In justification of the misapprehensions into which he has fallen, I readily admit that in my former letter there are isolated passages which warrant the construction upon which he has commented, and that it would have been better had these been more carefully and correctly expressed. I admit, farther, that I omitted all detailed or special recommendation of active medical treatment, and might thus erroneously be supposed to disparage it, even in cases where it is most required. But as my object in writing was to urge attention to *the neglected portion* of treatment, and *not to that which is already too actively followed,* viz., the use or abuse of drugs, I naturally dwelt on the former, and did not feel called upon to enter upon the consideration of the latter. Fearing, indeed, some such misconstruction, I warned you, towards the end of my former letter, that "I must trust to your good sense and right feeling not to give undue importance to any isolated or dubious expression which you may meet with, but *to adopt that meaning which is in harwony with the general spirit of my remarks*." Tried by this test, I still think I shall be found not to have undervalued active treatment where it is really called for by the state of the patient, and where the indications presented are judiciously fulfilled. In this respect your views, I believe, entirely coincide with my own.

And now for a parting word on homœopathy. *Commenting on your statements* of the success attendant on the homœopathic treatment of even severe diseases,as a sufficient and reasonable ground for instituting an inquiry into the alleged facts, I recommended as the safest and easiest preliminary step, that experiments should be made on the alleged power of certain medicines to produce *in healthy persons* forms of disease analogous to those in which they are given to cure; and as an example, I said, "Let experiments be made on a *sufficient number of* HEALTHY PERSONS with quinine or any other drug, to ascertain whether it really has the power ascribed to it, of exciting certain groups of symptoms IN A SOUND CONSTITUTION, and after carefully varying and repeating the experiments, faithfully record and publish the results;" by which means the principle of *similia similibus* may be proved or disproved beyond the possibility of a doubt. Not adverting to the real meaning of this proposition, Dr. Symonds remarks that "Dr. Andrew Combe tells us that" (in testing homœopathy) "we must carefully distinguish the principle of *similia similibus* from that of *infinitesimal doses,* and he suggests that we should test the former separately. But to do this we must begin an entirely new line of observations. And *what is to induce us to administer a scruple of jalap for a looseness, and a grain or two of opium for a lethargy?*" Dr. Symonds follows up this question by sundry exclamations, which need not be quoted, on the absurdity of such a course; but if he had correctly apprehended my proposition, he would have seen that it was nearly the reverse of that which he ascribes to me. As really stated by me, the question to be tried is, whether the medicine which *cures* (not causes) a looseness or lethargy, *will produce* the one or the other respectively *when administered to a person in health and of sound constitution.* Now, as he does not affirm that jalap *cures* a diarrhea, nor opium a lethargy, it is for himself and not for me to explain *why* we should be induced to prescribe the one for a looseness or the other for a lethargy. I candidly own that I cannot give him the information he asks, nor would the inquiry proposed by me be likely to lead to it.

I trust that I have now said enough to satisfy Dr. Symonds that the question really before the profession is by no means so frightful as he represents it to be, and that, contrary to his assumption, there is something approaching to unanimity among medical men, as to the folly and wickedness of "leaving diseases to follow their own course," when Nature has put so many means within our reach, by which they may be guided more safely and surely towards a successful issue. But while I think that Dr. Symonds has been rash in ascribing to some of the communications in your Review a meaning not warranted by either their substance or their spirit, I scarcely regret the publication of his letter, or of the analogous articles in the 'Medical Gazette' and other journals, as the discussion they have elicited will lead to good, by stirring up reflecting readers to the *better observation of Nature in both the study aud the treatment of disease*—a path which still seems to me to lead more directly to the land of promised improvement in our art than any other yet discovered. But not to trespass further on your patience, I remain,

My dear Sir,

Very sincerely yours,

ANDREW COMBE.

